# Genomic Characterization of Multidrug-Resistant and ESBL-Producing *Klebsiella pneumoniae* Isolated from Healthy Rabbits Intended for Human Consumption

**DOI:** 10.3390/microorganisms13081931

**Published:** 2025-08-18

**Authors:** Vanessa Silva, Manuela Caniça, Rani Rivière, Adriana Silva, Patrícia Poeta, Gilberto Igrejas

**Affiliations:** 1 LAQV-REQUIMTE, Department of Chemistry, NOVA School of Science and Technology, Universidade Nova de Lisboa, 1099-085 Caparica, Portugal; 2 Department of Genetics and Biotechnology, University of Trás-os-Montes and Alto Douro (UTAD), 5000-801 Vila Real, Portugal; 3 Functional Genomics and Proteomics Unit, University of Trás-os-Montes and Alto Douro (UTAD), 5000-801 Vila Real, Portugal; 4 Microbiology and Antibiotic Resistance Team (MicroART), Department of Veterinary Sciences, University of Trás-os-Montes and Alto Douro (UTAD), 5000-801 Vila Real, Portugal; 5 National Reference Laboratory of Antibiotic Resistances and Healthcare Associated Infections, Department of Infectious Diseases, National Institute of Health Dr. Ricardo Jorge, 1649-016 Lisbon, Portugal; manuela.canica@insa.min-saude.pt (M.C.); raniriviere1404@gmail.com (R.R.); 6 Centre for the Studies of Animal Science, Institute of Agrarian and Agri-Food Sciences and Technologies, University of Porto, 4099-002 Porto, Portugal; 7 Associate Laboratory for Animal and Veterinary Sciences (AL4AnimalS), 5000-801 Vila Real, Portugal; 8 CECAV—Veterinary and Animal Research Centre, University of Trás-os-Montes and Alto Douro, 5000-801 Vila Real, Portugal

**Keywords:** *Klebsiella pneumoniae*, rabbits, antimicrobial resistance, ESBL, whole-genome sequencing, One Health

## Abstract

*Klebsiella pneumoniae* is an important pathogen associated with multidrug resistance and virulence in both human and animal populations. While its prevalence and resistance patterns are well documented in clinical settings, data on *K. pneumoniae* in food-producing animals remain scarce. This study aimed to isolate and characterize multidrug-resistant *K. pneumoniae* strains from healthy rabbits raised for human consumption, with a focus on antimicrobial resistance genes, plasmid content, and associated mobile genetic elements. A total of 295 fecal samples were collected from rabbits across 20 commercial farms in northern Portugal. Isolates were confirmed using MALDI-TOF MS, tested for hypermucoviscosity, and subjected to antimicrobial susceptibility testing (EUCAST). Whole-genome sequencing (WGS) was performed to determine sequence types (STs), resistance genes, plasmids, and resistance determinants for metals and biocides. Six *K. pneumoniae* isolates were recovered, showing extensive antimicrobial resistance profiles, including ESBL genes such as *bla*_CTX-M-15_, *bla*_SHV-28_, and *bla*_TEM-1_. The most frequent ST was ST307. Multiple genes resistant to heavy metals were identified. Plasmid analysis revealed the presence of IncFII, IncN, and ColRNAI types. Network analysis showed clusters of genetically related isolates and highlighted shared resistance mechanisms. The presence of multidrug-resistant *K. pneumoniae* in healthy rabbits destined for human consumption underscores the zoonotic potential of this species and the need for surveillance in the animal–food–human interface. These findings contribute to a better understanding of resistance ecology in the context of One Health.

## 1. Introduction

*Klebsiella pneumoniae* is a Gram-negative, non-motile bacterium that plays an important role as a human pathogen. *K. pneumoniae* is an opportunistic pathogen that can cause a wide range of infections, most frequently pneumonia and urinary tract infections, followed by bacteremia, liver abscess, wound infections, and, less commonly, meningitis [1]. In recent years, *K. pneumoniae* has become a major global public health concern due to the emergence and rapid spread of extensively drug-resistant (XDR) strains, especially carbapenem-resistant *K. pneumoniae* (CRKP), as well as hypervirulent variants [1]. These resistant strains are part of the carbapenem-resistant Enterobacteriaceae (CRE) group and are particularly worrisome because they can resist treatment with carbapenems, which are broad-spectrum antibiotics often used as a last resort. The World Health Organization (WHO) has classified CRE as a “critical priority” for the development of new antibiotics [2,3]. The main mechanism behind carbapenem resistance in *K. pneumoniae* involves the production of carbapenemases, enzymes that degrade these antibiotics. These include class A carbapenemases (especially *K. pneumoniae* carbapenemase, KPC), class B metallo-β-lactamases (such as New Delhi metallo-β-lactamase, NDM), and some class D OXA-type enzymes [4].

Beyond clinical settings, *K. pneumoniae* can be found in diverse environments, including soil, water, and vegetation [5,6]. It is also capable of colonizing abiotic surfaces, such as medical equipment, and mucosal surfaces of humans—particularly the gastrointestinal tract and the oropharynx—from where it can potentially invade other tissues [7].

This bacterium has been isolated from a wide range of animal hosts, including both domestic and wild mammals, as well as insects and food products [6,8]. In animals, *K. pneumoniae* can cause diseases such as mastitis, pneumonia, and bacteremia [9].

Several studies have reported the possible zoonotic transmission of Enterobacterales, including *K. pneumoniae*, from companion animals, livestock, and wildlife [10,11]. Transmission can occur not only through human-to-human contact but also via close contact with animals, contaminated food, or environmental sources.

There is growing concern that livestock colonized by *K. pneumoniae* could act as reservoirs for antimicrobial resistance genes and virulence factors, which could eventually be transferred to human-associated strains [12]. While the molecular and epidemiological features of clinically important *K. pneumoniae* strains in humans are well characterized, much less is known about the population structure and behavior of *K. pneumoniae* in animals.

The presence of third-generation cephalosporin-, carbapenem-, and even colistin-resistant strains, along with hypervirulent variants, has been increasingly reported in both clinical and non-clinical settings [13,14]. However, the available studies rarely address the genetic background, resistance mechanisms, or plasmid content of *K. pneumoniae* isolates from food-producing animals. In *K. pneumoniae*, antimicrobial resistance is frequently mediated by mobile genetic elements carrying genes such as *bla*_CTX-M_, *bla*_SHV_, and *bla*_TEM_ (ESBLs), as well as carbapenemases like KPC, NDM, and OXA-48-like enzymes [15,16,17]. Additional mechanisms include plasmid-mediated quinolone resistance (*qnr*), aminoglycoside-modifying enzymes, and efflux pumps, often co-occurring with resistance genes to metals (*sil*, *pco*, *ars*) and biocides [18,19,20]. The combination of multiple determinants contributes to its multidrug-resistant phenotype.

Rabbit farming is particularly relevant in this context. Rabbits raised for meat on industrial farms show some of the highest rates of antimicrobial use among food-producing animals, which has led to worrying levels of antimicrobial resistance within the industry [21]. In 2018, the main exporters of rabbit meat were China, Spain, Hungary, France, and Italy, with major importers including Germany, Belgium, Portugal, Italy, and France [22]. The European Union is the second-largest global producer of rabbit meat, following China, and accounts for 93% of worldwide exports and imports [22]. Cultural and dietary preferences, especially in Mediterranean countries like Portugal, Spain, and Italy, are important drivers of rabbit meat consumption [23].

Nevertheless, studies on the prevalence of *K. pneumoniae* in meat rabbits are extremely scarce, despite the potential public health implications of antimicrobial-resistant strains in the food chain. Rabbits, as food-producing animals, may serve as reservoirs for clinically relevant resistance genes, which could be transmitted to humans through direct contact or food consumption [24,25]. Therefore, this study aimed to isolate *K. pneumoniae* from healthy rabbits intended for human consumption and to evaluate the antimicrobial resistance profiles, resistance genes, and genetic lineages of the isolates.

## 2. Materials and Methods

### 2.1. Sample Collection and Bacterial Isolation

A total of 295 fecal samples were collected from healthy rabbits, defined as animals without visible clinical signs of disease at the time of sampling, raised for human consumption, across 20 commercial farms located in the regions of Trás-os-Montes, Alto Tâmega, Douro, Ave, and Minho. On each farm, 15 samples were obtained from different areas within the rabbit hutch environment to ensure representative sampling across the facility.

An aliquot of 5 g from each fecal sample was homogenized in brain–heart infusion (BHI) broth and incubated aerobically at 37 °C for 24 h. Following enrichment, the cultures were streaked onto Simmons citrate agar (SCA) with 1% inositol (SCAI) and incubated at 40 °C for 48 h. One colony displaying morphological characteristics typical of *Klebsiella* spp. (yellow, dome-shaped colonies with a mucoid appearance) was selected and subcultured in HiCrome Klebsiella selective agar supplemented with Klebsiella Selective Supplement (HiMedia Laboratories, Maharashtra, India) to confirm the growth of purple–magenta colored colonies. Species-level identification of bacterial isolates was confirmed using matrix-assisted laser desorption/ionization time-of-flight mass spectrometry (MALDI-TOF MS) with the MALDI Biotyper^®^ system (Bruker Daltonik, Bremen, Germany).

### 2.2. String Test

The string test was conducted as previously described [26]. All isolates were cultured on blood agar plates incubated overnight at 37 °C. An inoculating loop was used to touch the colonies gently and lift them. A positive test was defined as a mucoid string >  5 mm in length observed visually.

### 2.3. Antimicrobial Susceptibility Testing

Antimicrobial susceptibility testing was performed using the Kirby–Bauer disk diffusion technique following the guidelines established by the European Committee on Antimicrobial Susceptibility Testing [27]. A total of 16 antibiotic agents (in µg/disk) were tested: gentamicin (10 µg), amikacin (30 µg), tobramycin (10 µg), streptomycin (10 µg), ampicillin (10 µg), amoxicillin–clavulanic acid (20 + 10 µg), cefoxitin (30 µg), ceftazidime (30 µg), aztreonam (30 µg), imipenem (10 µg), tetracycline (30 µg), nalidixic acid (30 µg), ciprofloxacin (5 µg), chloramphenicol (30 µg), and trimethoprim/sulfamethoxazole (1.25/23.75 µg). All antimicrobial disks were obtained from Oxoid Ltd. (Basingstoke, UK) and tested according to EUCAST guideline [27]. The presence of extended-spectrum β-lactamase (ESBL) production was phenotypically assessed using the double-disk synergy test (DDST), following EUCAST guidelines. Briefly, bacterial suspensions were adjusted to a 0.5 McFarland standard and inoculated onto Mueller–Hinton agar plates. A disk containing amoxicillin–clavulanic acid was placed at the center of the plate, and disks of third-generation cephalosporins (ceftazidime and cefotaxime) were placed 20 mm (center to center) away from the amoxicillin–clavulanic acid disk.

After incubation at 37 °C for 18–24 h, isolates showing a clear enhancement of the inhibition zone between the β-lactam antibiotic disk and the clavulanate disk (keyhole effect) were interpreted as ESBL producers.

### 2.4. Whole-Genome Sequencing

Whole-genome sequencing (WGS) was carried out on all *K. pneumoniae* isolates. Bacterial strains were initially cultured on MacConkey agar for differentiation and subsequently isolated on nutrient agar. Genomic DNA extraction was performed using the Magna Pure 96 system (Roche, Basel, Switzerland), following the manufacturer’s instructions. DNA concentrations were then quantified using a Qubit™ 4 fluorometer (Thermo Scientific, Waltham, MA, USA).

Sequencing libraries were prepared with the Nextera XT kit (Illumina, San Diego, CA, USA), and sequencing was conducted on an Illumina MiSeq platform, generating 150 bp paired-end reads. Raw sequencing data were subjected to bioinformatics analysis using a combination of tools for quality control, genome assembly, and annotation.

Read quality assessment and trimming were performed with FastQC (v0.11.5) and Trimmomatic (v0.38) [18], while genome completeness was evaluated using BUSCO (v5.5.0_cv1). De novo assembly, species confirmation, and sequence typing were conducted using INNUca (v4.2.2-02) (https://github.com/B-UMMI/INNUca; accessed in 30 December 2024). Species identification was further validated through average nucleotide identity (ANI) analysis, using FastANI (v1.33), by comparing assembled genomes against reference sequences retrieved from NCBI GenBank (https://www.ncbi.nlm.nih.gov/datasets/genome/?taxon=570; accessed in 30 December 2024). To detect antimicrobial resistance genes (ARGs), both abriTAMR (v1.0.14) and ABRicate (v1.0.1) were employed (http://github.com/tseemann/abricate; accessed in 30 December 2024). In ABRicate, several public databases were utilized, including ARG-ANNOT, ResFinder, CARD, NCBI, PlasmidFinder, and VFDB. Finally, multilocus sequence typing (MLST) was used to assign the isolates to their respective sequence types.

## 3. Results and Discussion

### 3.1. Prevalence and Phenotypic Resistance

Given the increasing concern regarding antimicrobial resistance in food-producing animals, the findings presented here provide important insights into the genomic background and resistance potential of *K. pneumoniae* strains isolated from rabbits raised for human consumption. Out of the 295 samples collected from healthy rabbits, only six (2%) were positive for *K. pneumoniae*, including two from the same farm. To the best of our knowledge, this is the first study investigating the prevalence and antimicrobial resistance of *K. pneumoniae* in healthy meat rabbits. Therefore, no direct comparisons can be made to determine whether the 2% prevalence observed in our study is considered low or not. Nevertheless, a study conducted on nasal samples from 147 wild rabbits reported a total of 14 *Klebsiella* isolates (9.5%), of which only 2 (1.4%) were identified as *K. pneumoniae*, a prevalence that is comparable to the 2% observed in our study [28]. In the same study, most of the *Klebsiella* isolates were susceptible to the majority of antibiotics tested. This contrasts with our findings, in which all isolates were classified as multidrug-resistant, displaying resistance to at least three different classes of antibiotics. This difference may be explained by the fact that wild animals, such as wild rabbits, are generally not exposed to antibiotics, whereas farmed rabbits are frequently treated with antimicrobial agents. Indeed, a study conducted in Italy by Agnoletti et al. reported that tetracyclines were the most commonly administered antibiotics in farmed rabbits, followed by polymyxins, sulfonamides, quinolones, and aminoglycosides [29].

**Figure 1:**
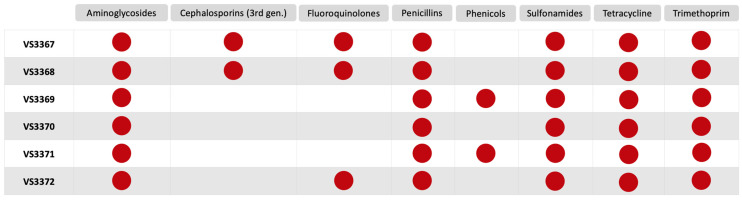
Phenotypic resistance profiles of six *K. pneumoniae* isolates grouped by antimicrobial class. Red dots indicate phenotypic resistance to at least one antibiotic within the corresponding class.

Phenotypically, in our study, all isolates demonstrated resistance to aminoglycosides, penicillins, trimethoprim/sulfamethoxazole, and tetracycline (Figure 1). Notably, isolates VS2267 and VS3368 exhibited resistance to third-generation cephalosporins and, together with isolate VS3372, also showed resistance to fluoroquinolones. In addition, two isolates (VS3369 and VS2271) displayed resistance to phenicols. The resistance profiles observed in our study are consistent with those reported in other studies conducted on farmed rabbits, although those studies focused on *Escherichia coli* rather than *K. pneumoniae* [30,31,32]. Phenotypic testing using the double-disk synergy test confirmed ESBL production in two isolates, showing a clear keyhole effect between third-generation cephalosporins and clavulanate.

### 3.2. Whole-Genome Sequencing

**Figure 2:**
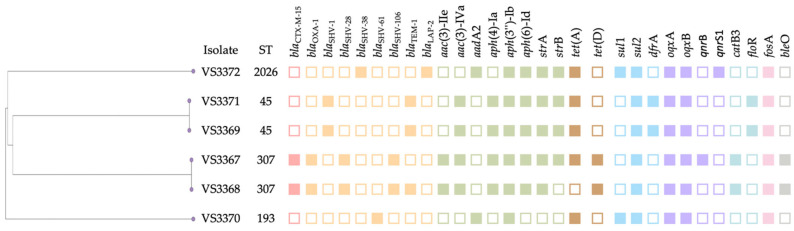
Dendrogram showing the genetic relatedness of the six *K. pneumoniae* isolates based on whole-genome data. Sequence types (STs) and the presence/absence of antimicrobial resistance genes are indicated alongside each isolate. Genes are grouped by antibiotic class and color-coded accordingly.

WGS was used to investigate the multilocus sequence types (MLSTs), as well as the presence of antimicrobial, biocide, and metal resistance genes, plasmid replicons, and virulence factors. The six *K. pneumoniae* isolates were assigned to four distinct sequence types: ST307 (*n* = 2), ST45 (*n* = 2), ST193, and ST2026 (Figure 2). Both ST307 isolates shared the same K and O loci (KL102), ST45 isolates were ascribed to KL62, ST193 to KL30, and ST2026 to KL2. Sequence type ST307 has been recognized as a globally emerging lineage of *K. pneumoniae*, associated with the dissemination of critical antimicrobial resistance (AMR) determinants, including ESBLs. This ST comprises multiple phylogenetic clades and has consistently been linked to the presence of the *bla*_CTX-M-15_ gene, a pattern that aligns with our findings [33,34,35]. In a comprehensive genomic study, Wyres et al. conducted whole-genome sequencing on 95 ST307 isolates carrying *bla*_CTX-M-15_ and *bla*_KPC_ genes from 11 countries, revealing that this clone likely emerged around 1994 [36]. Their analysis identified two major deep-branching lineages within ST307, one of which displayed widespread global distribution and was characterized by key mutations in the quinolone resistance-determining regions (QRDRs), specifically *gyr*A S83I and *par*C S80I. These same QRDR mutations were also detected in the ST307 isolates from our study, further reinforcing the lineage’s conserved resistance profile. Additionally, most ST307 isolates globally are reported to share identical capsular (K) and O-antigen loci, particularly KL102, a finding that was likewise observed in our isolates [37]. To our knowledge, no previous studies have investigated *K. pneumoniae* in healthy meat rabbits raised for human consumption. However, the presence of *K. pneumoniae* has been documented in pet rabbits, as reported in other contexts [6,38,39] suggesting that rabbits, regardless of their intended use, may serve as reservoirs for clinically relevant *K. pneumoniae* lineages.

Two of the isolates in our study were assigned to sequence type ST45. This lineage is frequently reported in clinical settings and appears to be relatively common among *K. pneumoniae* isolates from human patients, raising concerns about its zoonotic potential [40,41,42]. In Portugal, the country where our study was conducted, ST45 has been described among human clinical isolates, with most carrying the wzi101/K24 capsular type, a clone that, although not predominant, has been associated with multidrug-resistant (MDR) infections [43]. Interestingly, the ST45 isolates identified in our study harbored wzi149/KL2, suggesting potential genomic diversity within this lineage and highlighting the importance of monitoring non-human reservoirs. One isolate (VS3372) belonged to ST2026 and KL2 which has been previously found in *Tegillarca granosa* [44]. In the same study, *K. pneumoniae* from *T. granosa* carried 89 virulence-, 36 antibiotic-resistance-, and 70 heavy metal-tolerance-related genes.

In our study, *K. pneumoniae* isolates did not harbor any *K. pneumoniae* virulence-associated genes, as determined using the Kleborate database [45]. Notably, none of the hypervirulent-associated genes (including *rmpA2*, *iuc*, *iro*, and *allS*) were detected in our isolates, even though two isolates were of the capsular serotype K2 which has been strongly linked to hypervirulent *K. pneumoniae* strains [46]. These genomic findings are consistent with our phenotypic results since, despite exhibiting a markedly mucoid colony morphology, all isolates were negative for the string test. Although β-lactam antibiotics are reportedly rarely used in rabbits due to their association with drug-induced diarrhea, two *K. pneumoniae* isolates (VS3367 and VS3368) in our study were found to carry genes conferring resistance to cefotaxime, indicating an ESBL-positive profile [29]. Indeed, both isolates were phenotypically positive for the double-disk synergy for ESBL detection and carried the *bla*_CTX-M-15_ gene which is consistent with other studies that showed that the gene is strongly associated with ST307 [33,34,35]. These two isolates also carried *bla*_OXA-1_, *bla*_SHV-28_, and *bla*_SHV-106_. The *bla*_OXA-1_ gene is not classified as an ESBL on its own but it contributes to β-lactam resistance, particularly when co-occurring with other β-lactamase genes such as *bla*_CTX-M-15_ [47,48]. Furthermore, a study analyzed 2845 *bla*_OXA-1_ carrying *K. pneumoniae* and reported that ST11 was the predominant genetic lineage among these isolates, followed by ST307 [48]. In both ST307 isolates, several resistance genes were found to be associated with mobile genetic elements. Specifically, *qnr*B1 was linked to Tn5403, *aac* (3)-IV and *aph* (4)-Ia to ISEc59, *dfr*A14 to IS6100, and *bla*_CTX-M-15_ to ISEc9. Both isolates also harbored well-characterized chromosomal mutations associated with fluoroquinolone resistance, namely *gyr*A S83I and *par*C S80I. These mutations occur within the quinolone resistance-determining regions (QRDRs) and are known to significantly reduce susceptibility to fluoroquinolones [49]. In addition, both ST307 isolates carried *bla*_SHV-28_ and *bla*_SHV-106_, both of which are variants of the SHV β-lactamase family. Both SHV-28 and SHV-106 have been associated with ESBL activity and resistance to third-generation cephalosporins [50,51,52]. These two isolates were also the only ones in which the *tet* (D), *cat*B3, and *ble*O genes were identified, further supporting their unique resistance profiles. *cat*B3 encodes a chloramphenicol acetyltransferase, mediating resistance to phenicols, particularly chloramphenicol and florfenicol [53]. The *ble*O gene provides resistance to bleomycin, an antitumor glycopeptide antibiotic not typically used in veterinary medicine [54]. The *tet* (D) gene confers resistance to tetracyclines through an efflux pump mechanism and is less commonly reported than other tetracycline resistance genes such as *tet* (A) [55]. The *tet* (A) gene, which also encodes an efflux pump responsible for tetracycline resistance, was identified in all isolates except one, indicating a high prevalence of this resistance determinant among the *K. pneumoniae* strains analyzed [55]. The widespread presence of *tet* (A) may reflect the selective pressure exerted by the use of tetracyclines in rabbit farming, as these antibiotics are commonly administered in intensive production systems for prophylactic or metaphylactic purposes [29]. The study of Agnoletti et al. has shown that polymixins are the most consumed antibiotics in rabbit farming, followed by tetracylines, pleuromutilins, and sulfonamides [29]. However, none of the isolates in our study exhibited resistance to colistin. All six *K. pneumoniae* isolates carried genes conferring resistance to aminoglycosides, with a diverse set of aminoglycoside-modifying enzymes detected. The most prevalent genes were *aph* (3″)-Ib and aph (6)-Id, both found in all isolates, followed by *str*A (n = 5) and *str*B (n = 4). Additionally, *aac* (3)-IVa and *aph* (4)-Ia were detected in four isolates each, while *aad*A2 and *aac* (3)-IIe were found in two isolates. The presence of multiple AME genes per isolate suggests additive or complementary resistance mechanisms, consistent with the observed multidrug-resistant phenotypes.

Notably, the two isolates recovered from the same farm (VS3371 and VS3369) belonged to the same sequence type (ST45) and exhibited highly similar resistomes, sharing all detected resistance genes. Despite originating from the same farm, the isolates were obtained from samples collected in distinct areas of the rabbit housing, suggesting environmental dissemination or clonal persistence within the facility. Both isolates also harbored the *flo*R and *bla*_SHV-1_ genes, which were not detected in any of the other isolates. *flo*R is particularly relevant in veterinary settings due to its association with florfenicol resistance, a drug commonly used in food-producing animals [56,57,58]. Interestingly, in both ST45 isolates, a large cluster of resistance genes, including *aph* (3″)-Ib, *aac* (3)-IV, *aph* (6)-Id, *flo*R, *aph* (4)-Ia, *bla*_TEM-1A_, and *sul*2, was located in association with the insertion sequence ISEc59 which belongs to IS6 family [59]. This configuration suggests the presence of a composite multidrug resistance cassette, likely mobilized as a unit, enabling the co-transfer of resistance to aminoglycosides, phenicols, β-lactams, and sulfonamides.

The ST2026 isolate (VS3372) exhibited a distinct resistance gene profile, carrying *bla*_SHV-38_, *bla*_LAP-2_, and *qnr*S1, none of which were detected in the other isolates. This isolate exhibited a complex resistome architecture, with multiple resistance genes co-located on known mobile genetic elements. In particular, the detection of Tn6196, ISKpn19, and insertion sequences ISVsa3/5, associated with genes conferring resistance to biocides, sulfonamides, fluoroquinolones, aminoglycosides, tetracyclines, and β-lactams, underscores the potential for horizontal co-transfer of multidrug resistance. The *bla*_LAP-2_ gene and *qnr*S1 were linked to the insertion sequence ISKpn19 which is in accordance with findings reported in other studies [60,61,62]. The *bla*_SHV-38_ gene had a 146V amino acid substitution, a mutation located near the catalytic region of β-lactamase, which is believed to cause minor structural alterations that enhance hydrolysis of imipenem, while not affecting meropenem [63]. Nevertheless, all isolates in our study were susceptible to carbapenems.

The ST193 isolate (VS3370), a sequence type for which limited data is available in the literature, exhibited the smallest resistome among the six, carrying only a few resistance genes, including *bla*_SHV-61_ with a 35Q amino acid substitution. Although the functional impact of this mutation remains unclear, such substitutions in SHV β-lactamases may influence substrate affinity or inhibitor susceptibility. The wzi275 KL30 capsular locus identified in this isolate is considered rare, with few reports available in the literature [64,65]. Its detection in this context contributes to the overall capsular diversity observed among the *K. pneumoniae* isolates and may reflect the limited circulation or underreporting of this capsular type in animal-associated strains.

A wide range of metal resistance genes was detected across the *K. pneumoniae* isolates, with the most extensive profiles found in the ST207 and ST2026 strains (Table 1). These included complete operons for silver (*sil*), copper (*pco*), and arsenic (*ars*) resistance, as well as the *fie*F gene involved in zinc homeostasis [66,67,68]. Notably, isolates VS3367 and VS3368 (ST207) and VS3372 (ST2026) harbored all detected metal resistance genes, suggesting exposure to environments with selective pressure from heavy metals. In contrast, VS3370 (ST193) encoded only *fieF*, highlighting the heterogeneity among isolates. The co-occurrence of these metal resistance determinants with antimicrobial resistance genes raises concerns about co-selection and persistence of multidrug-resistant strains in animal production environments [69,70]. The co-occurrence of complete *ars*, *pco*, and *sil* resistance operons in multiple isolates, especially those with expanded resistomes, may indicate potential co-selection pressure in farm environments [71,72]. These operons are known to occur on mobile genetic elements and can be linked to antimicrobial resistance genes, facilitating co-resistance [73,74]. The detection of *fie*F across all isolates also suggests conserved mechanisms for metal homeostasis, even in strains with otherwise limited metal resistance profiles [68]. Altogether, these findings support the hypothesis that non-antibiotic selective pressures in animal production may contribute to the emergence and maintenance of multidrug-resistant *K. pneumoniae*. The widespread detection of metal resistance genes may reflect selective pressures associated with common agricultural practices. In rabbit farming and other livestock production systems, copper and arsenic compounds have historically been used as feed additives and growth promoters, while silver-based agents are often applied as disinfectants [72,75,76]. Copper compounds (e.g., copper sulfate) remain authorized for use in rabbit diets, provided that regulatory maximum levels as defined by EU Regulation 2018/1039 are adhered to (e.g., up to 35 mg/kg in cattle feed and 150 mg/kg in piglets) [77,78,79]. EFSA considered copper sulfate pentahydrate safe for all food-producing species, including rabbits, when used within these limits [80]. Similarly, arsenic-based additives, such as arsanilic acid, were previously used in livestock feed formulations [81,82]. These environmental exposures can select for bacteria harboring metal resistance genes, which are frequently located on the same mobile genetic elements as antimicrobial resistance genes. This genetic co-localization promotes co-selection, contributing to the persistence and dissemination of multidrug-resistant bacteria, even in the absence of direct antibiotic use [70,83,84,85]. The detection of these metal resistance operons in *K. pneumoniae* isolates from healthy meat rabbits thus reflects a complex ecological interplay between farming practices and antimicrobial resistance dynamics, highlighting the importance of considering non-antibiotic selective pressures in food-producing animal systems.

**Figure 3:**
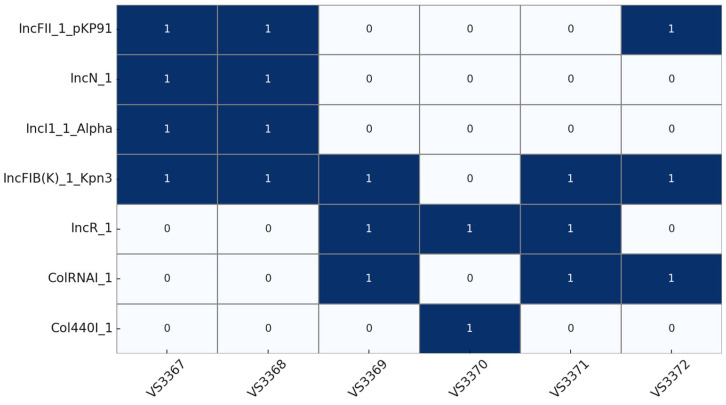
Distribution of plasmid replicon types detected in each *K. pneumoniae* isolate based on whole-genome sequencing. Dark blue cells indicate presence (1), and white cells indicate absence (0) of the corresponding replicon.

Seven different plasmid replicons were detected among *K. pneumoniae* isolates (Figure 3). Plasmids IncFII_1_pKP91, IncFIB (K)_1_Kpn3, IncN_1, and IncI1_1_Alpha were detected in the two ST307 isolates. The presence of IncFII and IncFIB (K) is consistent with the global plasmidome associated with ST307, a high-risk clone typically carrying *bla*_CTX-M-15_*, qnr*B, and *tet* (A) [36,86]. Plasmids belonging to the IncN group are commonly found in and mobilized between members of Enterobacterales while IncI1-I plasmids have been predominantly associated with ESBL-producers from animals and humans, facilitating inter-reservoir transfer [87,88,89]. In contrast, the ST45 isolates carried IncFIB (K)_1_Kpn3, IncR_1, and ColRNAI_1, reflecting a distinct but still diverse plasmid background. Plasmids of the IncF incompatibility group are among the most commonly reported in bacterial isolates from both human and animal sources. These plasmids are frequently linked to antimicrobial resistance genes and play a key role in the dissemination of resistance, particularly to quinolones, aminoglycosides, extended-spectrum β-lactams (ESBLs), and carbapenem [73]. The ST193 isolate (genetically less complex and with the smallest resistome) harbored only IncR_1 and Col440I_1. The ST2026 isolate presented a combination of IncFII_1_pKP91, IncFIB (K)_1_Kpn3, and ColRNAI_1, mirroring the ST307 plasmidome and supporting the idea of shared genetic features despite sequence type divergence. Interestingly, many of the plasmids analyzed in this study contained conserved regions encompassing the *sil* and *pco* operons, suggesting that this genetic arrangement may be a recurrent feature in *K. pneumoniae* plasmids of the IncFIB incompatibility group. Collectively, these findings suggest that specific plasmid combinations may contribute to the observed differences in resistome size and diversity, and that food-producing animals could serve as reservoirs for plasmids of clinical relevance.

**Figure 4:**
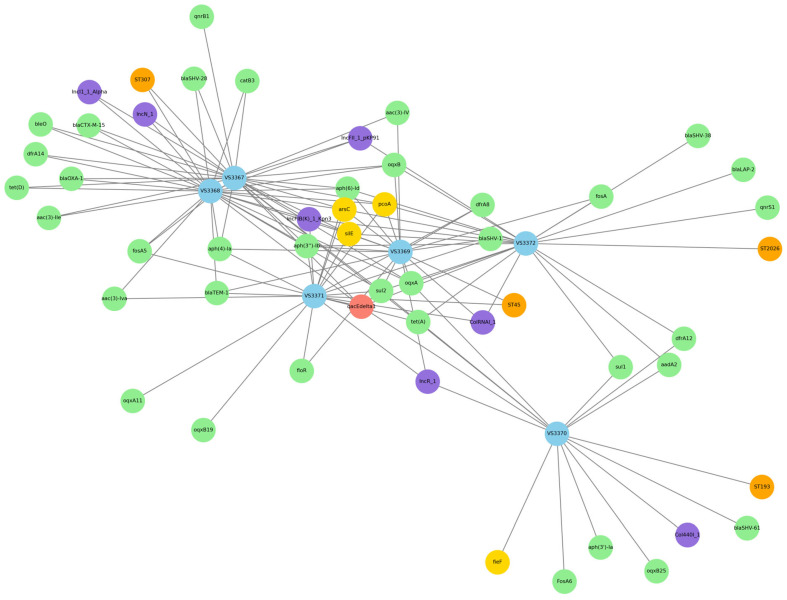
Network representation of *K. pneumoniae* isolates showing relationships among antimicrobial resistance genes (green nodes), plasmids (purple), metal resistance genes (yellow), biocide resistance genes (red), and sequence types (STs, orange). Isolates are represented in blue. Edges indicate co-occurrence in the same isolate, and node proximity reflects functional similarity or shared profiles. The network highlights the genetic complexity and interconnectivity among resistance determinants.

A comprehensive network analysis was constructed to visualize the interrelationships among sequence types, antimicrobial resistance genes, plasmids, metal resistance genes, and biocide resistance genes found in *K. pneumoniae* isolates (Figure 4). The network revealed distinct clusters of genetically related isolates, such as VS3367 and VS3368, both associated with ST307 and sharing an identical AMR and plasmid profile. In contrast, isolate VS3370 exhibited a more divergent profile, featuring unique resistance genes (*bla*_SHV-61_, *sul*1) and the plasmid Col440I_1. The co-occurrence of multiple resistance elements in individual isolates underlines the potential for horizontal gene transfer and highlights the role of healthy food-producing animals as reservoirs of clinically relevant resistance determinants.

## 4. Conclusions

This study represents the first report of *K. pneumoniae* isolated from healthy meat rabbits raised for human consumption. Despite the low prevalence (2%), all six isolates were multidrug-resistant and harbored diverse antimicrobial resistance genes, metal resistance operons, and plasmid replicons. Notably, two isolates belonged to the globally disseminated ST307 clone, carrying *bla*_CTX-M-15_, *bla*_OXA-1_, and multiple resistance genes associated with mobile genetic elements. The presence of resistance to clinically relevant antibiotics, including third-generation cephalosporins and fluoroquinolones, as well as extensive metal resistance determinants (arsenic, copper, silver) raises concerns about co-selection pressures within rabbit farming systems.

The detection of conjugative plasmids frequently implicated in zoonotic and clinical antimicrobial resistance gene dissemination, such as IncFII, IncN, and IncI1, further highlights the potential public health implications. These findings underscore the importance of monitoring *K. pneumoniae* in non-traditional food animal species and emphasize the need to consider both antibiotic and non-antibiotic selective forces, such as heavy metals, in the ecology and evolution of antimicrobial resistance.

## Figures and Tables

**Table 1:** Presence of metal resistance genes detected in each *K. pneumoniae* isolate. Green plus signs (+) indicate the presence of the gene; red minus signs (−) indicate its absence. *sil* genes are associated with silver resistance, *pco* genes with copper resistance, *ars* genes with arsenic resistance, and *fieF* with iron transport.

Isolate	*fieF*	*silE*	*silS*	*silR*	*silC*	*silF*	*silB*	*silA*	*silP*	*pcoA*	*pcoB*	*pcoC*	*pcoD*	*pcoR*	*pcoS*	*pcoE*	*arsC*	*arsB*	*arsA*	*arsD*	*arsR*
VS3367	+	+	+	+	+	+	+	+	+	+	+	+	+	+	+	+	+	+	+	+	+
VS3368	+	+	+	+	+	+	+	+	+	+	+	+	+	+	+	+	+	+	+	+	+
VS3369	+	+	+	+	+	+	+	+	+	+	+	+	+	+	+	−	+	+	+	+	+
VS3370	+	−	−	−	−	−	−	−	−	−	−	−	−	−	−	−	−	−	−	−	−
VS3371	+	+	+	+	+	+	+	+	+	+	+	+	+	+	+	−	+	+	+	+	+
VS3372	+	+	+	+	+	+	+	+	+	+	+	+	+	+	+	+	+	+	+	+	+

## Data Availability

The original contributions presented in this study are included in the article. Further inquiries can be directed to the corresponding authors.
